# The effect of electromagnetic field on sleep of patients with nocturia

**DOI:** 10.1097/MD.0000000000029129

**Published:** 2022-08-12

**Authors:** Shin-Hong Chen, Wei-Chih Chin, Yu-Shu Huang, Leonard S. Chuech, Chang-Min Lin, Chin-Pang Lee, Huang-Li Lin, I Tang, Ting-Chun Yeh

**Affiliations:** a Division of Urology, Taiwan Adventist Hospital, Taipei, Taiwan; b Division of Pediatric Psychiatry and Sleep Center, Chang Gung Memorial Hospital, Taoyuan, Taiwan; c College of Medicine, Chang Gung University, Taoyuan, Taiwan.

**Keywords:** American Urological Association Symptom Score, electromagnetic field, insomnia, nocturia, Pittsburgh sleep quality index

## Abstract

**Introduction:**

Accumulated studies revealed that electromagnetic field can affect human brain and sleep. We explored the effectiveness of electromagnetic field [Schumann resonance (SR)] on nocturia symptoms, quality of life, and sleep in patients with nocturia.

**Methods:**

This is a randomized, open-label, and active-controlled study, in which 35 participants were randomized into 2 groups. Group A received oxybutynin and the SR device for 12 weeks, while the active-control group received only the medication. We followed these patients every 4 weeks with a number of questionnaires, including the Pittsburgh sleep quality index (PSQI) and Epworth sleepiness scale (ESS) for sleep, the American Urological Association Symptom Score (AUASS) for nocturia symptoms, and the Nocturia-Quality-of-Life-questionnaire (N-QOL) for quality of life. Descriptive statistics, pair t-tests, Chi-squared tests, and repeated measures were applied for data analysis.

**Results:**

No significant difference was found in the demographic data between the 2 groups. The AUASS, N-QOL, PSQI, and ESS total scores were significantly improved in the SR-sleep-device group (*P* < .001, *P* = .005, *P* < .001, *P* = .001) after treatment, but no significant change was found in the active-control group. Several variables of AUASS in the SR-sleep-device group were significantly improved, especially streaming and sleeping (both *P* = .001), and subjective sleep quality and sleep efficiency also demonstrated significant improvement (both *P* < .001).

**Conclusions:**

Our study revealed that electromagnetic field (SR) as an add-on can improve not only sleep and quality of life but also nocturia symptoms in patients with nocturia. These findings suggest that SR can be effective for sleep disturbance secondary to physical disease, which can be a new application of the electromagnetic field.

## 1. Introduction

The International Continence Society defines nocturia as waking during the night at least once to urinate.^[1]^ The prevalence is high in both genders and increases with age. Up to 61% of elderly women and up to 59% of elderly men report 2 or more voids per night.^[2]^ Furthermore, 5% to 15% aged 20 to 50 years, 20% to 30% aged 50 to 70 years, and 10% to 50% aged ≥70 years had >2 nocturia episodes per night.^[3]^ This condition has profound influences on both health and quality of life (QoL). More than 60% of men and women reported that nocturia had a negative effect on their QoL, while a systematic review of the relationship between nocturia and mood indicated that nocturia and depression/anxiety can be strongly associated.^[4]^

Nocturia is a cause of sleep disturbance and can impair patients’ functioning, QoL, and health.^[5]^ Sleep fragmentation and disruption further result in daytime sleepiness, tiredness, mood changes, and such cognitive dysfunction as poor concentration and performance.^[5,6]^ The most common treatment for nocturia is pharmacological therapy. However, with the exception of desmopressin for the treatment of nocturnal polyuria, the International Consultations of Urological Diseases Committee classified the strength of most nocturia medications as low.^[7]^ Patients with nocturia can still be disturbed by nocturia and related sleep disturbances despite taking such medications as antimuscarinic agents. Moreover, side effects of these medications include dry mouth and constipation, which can be intolerable and further limit its usage or dosage titration.

Pharmacological treatment for insomnia, such as benzodiazepines, is commonly prescribed clinically for these patients, but these medications can lead to tolerance and dependence. Their sedative effects can also increase the risk of falling, especially in the elderly and those with nocturia who need to use the toilet at night. Nonpharmacological treatments like cognitive behavioral therapy for insomnia (CBTi) and exercise may reduce nocturia and improve sleep,^[8]^ but such treatment is often not accessible, and the inconvenience and patient compliance remain to be key issues in the implementation of such treatments. Moreover, the efficacy of CBTi for the senior population has less scientific evidence than for the adult population. Therefore, exploring the effectiveness of other and more emerging nonpharmaceutical treatments for insomnia is important.^[9]^

A special discovery over the past 2 decades is that ambient electromagnetic fluctuations, such as geomagnetic activity, can influence humans’ physiology, psychology, and behavior,^[10]^ and studies have shown moderate strength correlations between increased geomagnetic activity and behavioral inferences of cerebral activity.^[11]^ Ghione et al^[12]^ found positive associations between geomagnetic activity and systolic/diastolic blood pressure. Furthermore, Burch et al^[13]^ reported that increasing geomagnetic activity with elevated 60 Hz MF is associated with reduced nocturnal excretion of a melatonin metabolite in humans, meaning that electromagnetic fields (EMFs) affect the brain and sleep.

Wang et al^[14]^ reported a strong, specific human brain response to ecologically relevant rotations of Earth-strength magnetic fields. Following geomagnetic stimulation, electroencephalography (EEG) showed a drop in amplitude of alpha oscillations (8–13 Hz) in a repeatable manner. In 1954, Winfried Otto Schumann reported a natural extremely low-frequency field (7.83 Hz) called the Schumann resonance (SR) frequency in Earth’s atmosphere that propagates EMF waves.^[15]^ Its peak intensity can be detected at ~8 Hz, along with its harmonics with a lower intensity at 14, 20, 26, 33, 39, and 45 Hz due to frequency-related, ionospheric propagation loss.^[16]^ SR can be found within both global human quantitative electroencephalographic activity and Earth-ionosphere activity, suggesting a causal relationship.^[17]^

With such accumulated evidence, extremely low-frequency EMF-SR has the potential to influence and improve human sleep. Our previous study demonstrated that the SR sleep device can improve nighttime sleep and daytime sleepiness through both objective sleep measurements and subjective sleep questionnaires in patients with primary insomnia (Y.S. Huang, I. Tan, W. Chin, et al. unpublished data). However, its effectiveness for patients with other sleep problem remains unknown. Therefore, we designed this randomized, open-label study and further applied the SR sleep device to patients with nocturia to evaluate the role of EMF and its application in patients with physical diseases and related sleep disturbance.

## 2. Material and Methods

This is a randomized, open-label and active-controlled study. Patients who met the inclusion criteria were recruited from the Urology clinic of the Taiwan Adventist Hospital, and then equally divided into group A and group B. The participants in group A received pharmacological treatment plus the SR sleep device per for 12 weeks, while those in group B (the active control group) received only pharmacological treatment. Both groups were evaluated using the Pittsburgh Sleep Quality Index (PSQI), Epworth Sleepiness Scale (ESS), American Urological Association Symptom Score (AUASS), and Nocturia QoL questionnaire (N-QOL) throughout the 12 weeks of intervention.

This study was approved by the institutional review board of the Taiwan Adventist Hospital (No. 106-E-32), and all participants signed the informed consent form.

### 2.1. Participants

Thirty-seven participants were enrolled after screening, but 2 dropped out because they could not attend regular follow-up. As a result, a total of 35 participants completed the study, 20 in the SR sleep device group A and 15 in the active control group B.

#### 2.1.1. Inclusion criteria.

Participants had to meet all of the following criteria: male or female aged >20 years old, nocturia for >4 weeks, willing to sign the informed consent form, and capable of cooperating to take medication and/or use the sleep device daily during the study.

#### 2.1.2. Exclusion criteria.

Participants who met any of the following exclusion criteria were not included in the study: patients with a pacemaker or cardiac monitor, patients with a clinically significant or unstable medical or surgical condition, such as heart diseases, metabolic diseases, renal insufficiency, hepatic diseases, urologic cancers, etc, patients with severe mental disorders, such as substance use disorders, schizophrenia, major depression, severe anxiety disorder, etc, patients with severe neurological diseases, such as Parkinson disease, stroke, brain injury, etc, patients with severe sleep disorders, such as severe sleep apnea syndrome, periodic limb movement disorder (PLMD), narcolepsy, etc, male patients with serum prostatic specific antigen levels >20 ng/mL, evidence of acute urinary infection by urine analysis, patients with polyuria, patients who have received oxybutynin or any pharmacological treatments for insomnia within 14 days before randomization, patients who were pregnant or breast feeding, and patients who were unable to commit to the scheduled follow-up or sleep hygiene.

### 2.2. Study protocol

**Figure 1.**
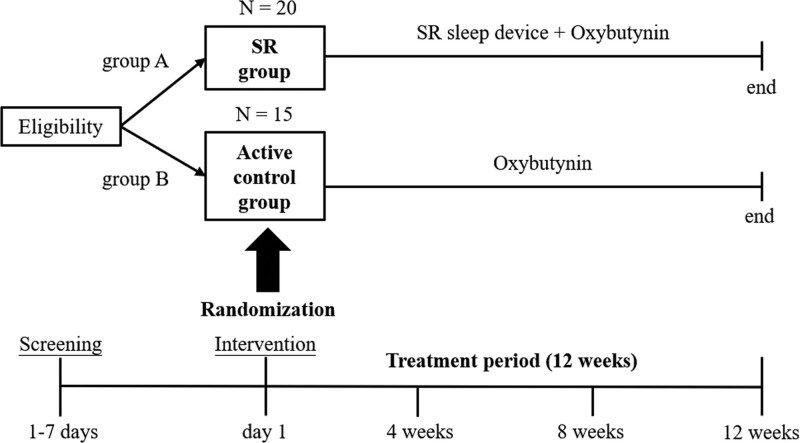
The flow chart of the study. SR = Schumann resonance.

A thorough clinical evaluation including history taking, physical examination, blood tests, and urine analysis was performed as screening. The patients also needed to keep voiding diaries. After screening, participants were randomly assigned to group A (the SR device group) or group B (the active control group). Group A received both the pharmacological treatment (oxybutynin 5 mg, PO, QD) and the SR sleep device for 12 weeks, while group B received only the pharmacological treatment (oxybutynin 5 mg, PO, QD) for 12 weeks. Any adverse effects of the interventions were recorded during follow-up. Participants were not allowed to take medication for insomnia and had to maintain previous treatment or medication for nocturia during the study to evaluate the effectiveness of interventions of the study from baseline. Figure 1 shows the study flow chart, and each participant was required to undergo a screening visit with subsequent follow-up visits every 4 weeks from baseline, for a total of 5 visits.

### 2.3. Interventions

#### 2.3.1. SR sleep device.

**Figure 2.**
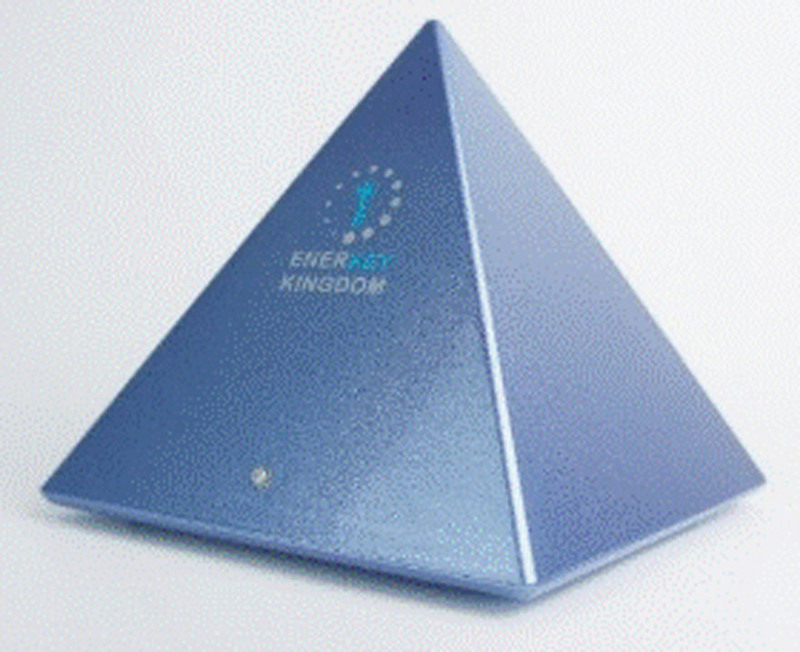
The SR sleep device.^18^ SR = Schumann resonance.

The SR sleep device (Enerkey Kingdom Sleep device) used in this study was designed by Professor Ling-Sheng Zhang from National Cheng Kung University, Taiwan (Fig. 2; Y.S. Huang, I. Tan, W. Chin, et al. unpublished data). This noninvasive health instrument can generate the low frequency of the SR frequency (7.83 Hz) wave and obtained a Taiwan Patent (No. TW M530656U) on May 4, 2016 (Fig. 2; Y.S. Huang, I. Tan, W. Chin, et al. unpublished data). Once the SR sleep device is turned on, it steadily outputs the composite waves of the SR frequency (7.83 Hz) wave, theta wave, and delta wave. Participants were asked to use the device (place it next to the bed facing the head, turn it on about 1 hour before going to bed, and turn it off after getting up the next day) and keep record in a sleep diary every night for 12 weeks

#### 2.3.2. Oxybutynin.

Oxybutynin is an antimuscarinic and antispasmodic medication that can relax the bladder muscles to decrease urgency and frequency, as well as symptoms of nocturia.^[18]^ In this study, the participants of both groups took oxybutynin 5 mg orally once per day.

#### 2.3.3. Subjective questionnaires for evaluation of sleep and nocturia.

Participants of both groups received subjective questionnaires to evaluate their sleep and nocturia. All questionnaires were administered at baseline and at each follow-up every 4 weeks during the study period.

##### 2.3.3.1. Pittsburgh sleep quality index.

The PSQI is a self-rated questionnaire for assessing sleep quality. It consists of 19 items and can be calculated into 7 component scores (0–3), with higher scores indicating worse sleep quality.^[19,20]^

##### 2.3.3.2. Epworth sleepiness scale.

The ESS consists of 8 items (scored from 0 to 3) and is used to assess daytime sleepiness.^[21]^ Higher scores indicated more daytime sleepiness.

##### 2.3.3.3. American Urological Association Symptom Score.

The AUASS is a questionnaire with 7 items covering frequency, nocturia, weak urinary stream, hesitancy, intermittence, incomplete emptying, and urgency. It can evaluate the symptoms severity of prostate hypertrophy and has been shown to be clinically sensible, reliable, valid, and reproducible.^[22]^

##### 2.3.3.4. Nocturia Quality - of-Life questionnaire.

The N-QOL is a patient-based questionnaire developed by Hunt et al.^[23]^ It evaluates the impact of nocturia on a patient’s QoL and consists of 31 items (scored from 0 to 4) with a 5-point Likert-like response scale. Items cover psychological distress, sleep, interference with habits, impact on relationships, limitations on activities and travel, physical energy, concentration, and safety. Higher scores indicate better quality of life.

### 2.4. Statistical analysis

We adopted intention-to-treat (ITT) analysis for all randomized participants who received intervention and had at least 1 follow-up evaluation, regardless of their compliance with the protocol or their study eligibility. Variables were presented as either mean ± standard deviation or frequency. We also analyzed the demographic data of the 2 groups using pair t-test and Chi-squared test. Comparisons of pretherapy and posttherapy data were analyzed with repeated measures, ANOVA, post hoc with Bonferroni, and independent sample t-test.

## 3. Results

Table 1 shows the demographic data of the 2 groups at baseline. Among the 35 participants with nocturia, 82.86% were male, with an average age of 62.09 ± 13.26 years. No significant difference was found in age, gender, body weight, body height, body mass index, hypertension, or diabetes mellitus between the 2 groups. Furthermore, we observed no significant difference in the baseline scores of AUASS, N-QOL, PSQI, or ESS.

**Table 1:** Demographic data of the SR device group and the active control group.

	Total (n = 35)	Group A (n = 20)	Group B (n = 15)	*P* value
Age, y	62.09 ± 13.26	65.40 ± 11.23	57.67 ± 15.97	.102
Gender, male, n (%)	29 (82.86%)	15 (75.00%)	14 (93.30%)	.154
Weight	64.71 ± 8.85	63.19 ± 8.03	66.73 ± 9.95	.253
Height	166.49 ± 7.41	166.06 ± 7.55	167.07 ± 7.23	.694
BMI	23.34 ± 2.88	22.96 ± 2.96	23.84 ± 2.77	.379
Hypertension, n (%)	10 (28.57%)	6 (30.00%)	4 (26.67%)	.829
Diabetes mellitus, n (%)	7 (20.00%)	5 (25.00%)	2 (13.33%)	.393
AUASS (baseline)	14.89 ± 6.56	16.40 ± 6.27	12.87 ± 6.94	.124
N-QOL (baseline)	29.6 ± 8.67	27.50 ± 8.74	32.40 ± 8.57	.107
PSQI (baseline)	8.80 ± 4.06	9.45 ± 3.97	7.93 ± 4.17	.281
ESS (baseline)	8.37 ± 4.31	9.25 ± 4.04	7.20 ± 4.68	.174

**Figure 3.**
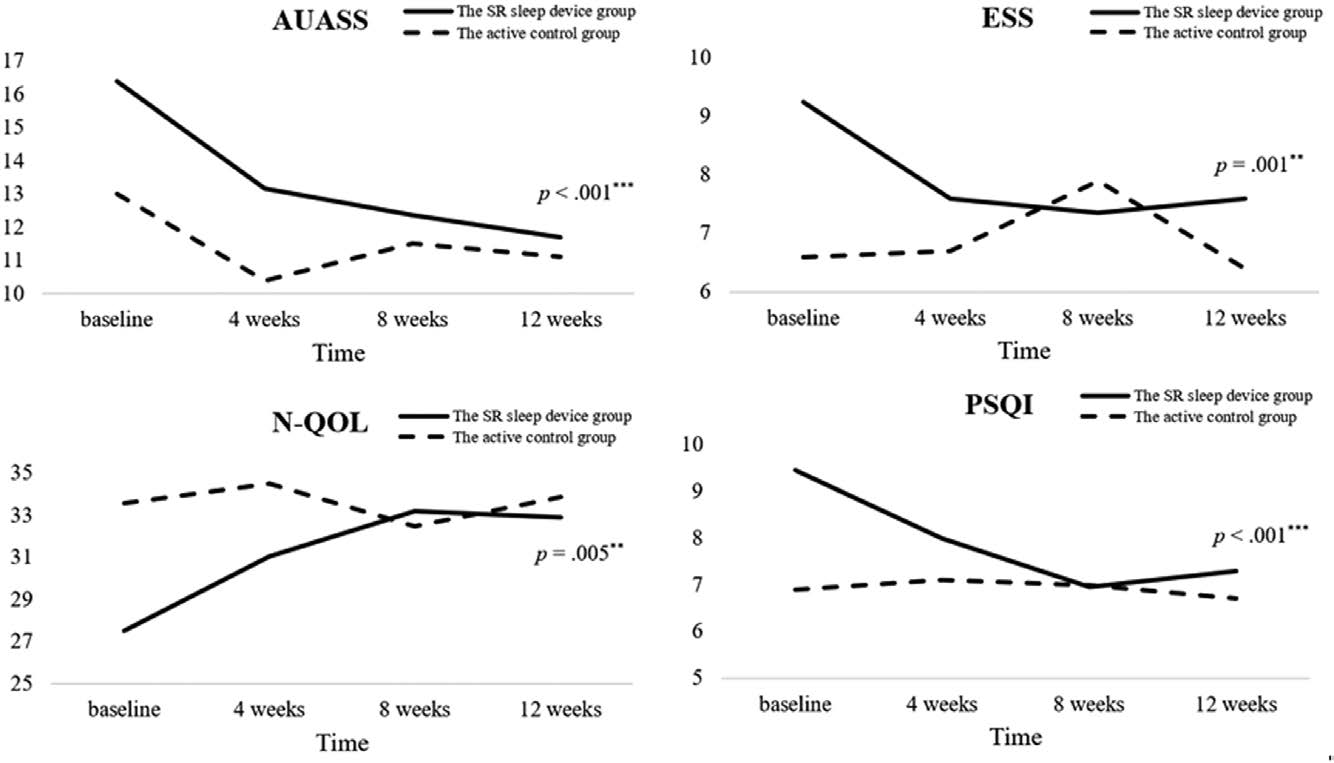
Changes of questionnaire data of the 2 groups during follow-up. AUASS = American Urological Association Symptom Score, ESS = Epworth sleepiness scale, N-QOL = nocturia quality-of-Life questionnaire, PSQI = Pittsburgh sleep quality index, SR = Schumann resonance.

Table 2 and Figure 3 show the questionnaire data collected from the 2 groups during follow-up. The total scores of AUASS, N-QOL, PSQI, and ESS were all significantly improved in group A, the SR device group, (*P* < .001, *P* = .005, *P* < .001, *P* = .001), but no significant changes were found in group B, the active control group (Fig. 3A–D). All significant improvements of group A could be found after 4 to 8 weeks of treatment. Although no significant group difference was found in AUASS, N-QOL, PSQI and ESS at the 12-week follow-up (*P* = .881, .825, .901, .937), we found significant more improvement of ESS (*P*2 = .032) and trends of more improvement of N-QOL and PSQI (*P*2 = .092, .054) in the experimental group.

**Table 2:** Total scores of questionnaires of the SR device group and the active control group during follow-up.

	Group A					Group B					*P*2
(M ± SD)	Baseline	4 wks	8 wks	12 wks	*P*1 (post hoc)	Baseline	4 wks	8 wks	12 wks	*P*1	
AUASS	16.40 ± 6.27	13.15 ± 5.70	12.35 ± 6.45	11.70 ± 5.91	<.001*** (1 > 2, 3, 4)	12.87 ± 6.94	11.13 ± 5.17	11.60 ± 5.30	11.40 ± 5.67	.513	.101
N-QOL	27.50 ± 8.74	31.05 ± 8.17	33.20 ± 8.33	32.90 ± 8.12	.005** (1 < 3, 4)	32.40 ± 8.57	33.07 ± 8.38	32.93 ± 8.57	33.53 ± 8.62	.869	.092
PSQI	9.45 ± 3.97	8.00 ± 2.94	6.95 ± 3.20	7.30 ± 3.31	<.001*** (1 > 3, 4)	7.93 ± 4.17	8.00 ± 4.64	7.80 ± 4.83	7.47 ± 4.55	.705	.054
ESS	9.25 ± 4.04	7.60 ± 4.37	7.35 ± 4.17	7.60 ± 4.28	.001** (1 > 2, 3, 4)	7.20 ± 4.68	7.60 ± 5.33	8.20 ± 5.77	7.47 ± 5.62	.520	.032*

We further analyzed the changes of variables of AUASS and PSQI during follow-up by repeated measures ANOVA. We found that incomplete emptying, urgency, streaming, and sleeping of AUASS in group A were all significantly improved (*P* = .04, .046, .001, and .001 respectively), and post hoc analysis showed significant improvement in streaming after 4-week intervention (1 > 3, 4; 2 > 4) and sleeping after initiating intervention (1 > 2, 3, 4). No significant changes were found in the variables of AUASS in group B (Table 3). Table 4 shows the analysis of variables of PSQI. Subjective sleep quality, sleep latency, and sleep efficiency of the SR sleep device group demonstrated significant improvement during follow-up (*P* < .001, *P* = .008, and *P* < .001 respectively), and post hoc analysis showed significant improvement in subjective sleep quality and sleep efficiency after initiating intervention (1 > 2, 3, 4). None of the PSQI variables showed significant improvement in the active control group, indicating that the medication alone (oxybutynin 5 mg once per day) offered limited improvement to the sleep of patients with nocturia during the 3-month study treatment.

**Table 3:** Changes of AUASS of the SR device group and the active control group during follow-up.

	Group A					Group B				
(M ± SD)	Baseline	4 wks	8 wks	12 wks	*P* value (post hoc)	Baseline	4 wks	8 wks	12 wks	*P* value
Incomplete emptying	2.30 ± 1.56	1.95 ± 1.23	1.80 ± 1.44	1.70 ± 1.13	.040*	1.93 ± 1.44	1.60 ± 1.30	1.93 ± 1.44	1.93 ± 1.28	.503
Frequency	3.35 ± 1.35	2.85 ± 1.42	3.05 ± 1.36	2.80 ± 1.20	.057	2.20 ± 1.21	2.00 ± 1.07	1.87 ± 0.74	1.80 ± 1.15	.435
Intermittency	1.40 ± 1.64	1.40 ± 1.47	1.30 ± 1.34	1.15 ± 1.31	.370	1.87 ± 1.25	1.53 ± 0.92	1.67 ± 1.40	1.67 ± 1.18	.792
Urgency	2.50 ± 1.61	1.85 ± 1.46	1.75 ± 1.68	1.65 ± 1.46	.046*	1.47 ± 1.64	1.07 ± 0.88	1.13 ± 0.99	0.93 ± 0.80	.279
Weak stream	2.70 ± 1.78	2.30 ± 1.42	1.60 ± 1.35	1.50 ± 1.24	.001** (1 > 3, 4; 2 > 4)	1.73 ± 1.16	1.53 ± 0.92	1.60 ± 1.12	1.73 ± 1.39	.868
Straining	0.90 ± 0.97	0.45 ± 0.60	0.60 ± 0.75	0.65 ± 0.93	.063	1.47 ± 1.46	1.27 ± 1.10	1.27 ± 0.96	1.13 ± 0.83	.589
Sleeping	3.25 ± 1.16	2.35 ± 1.18	2.25 ± 1.07	2.25 ± 1.29	.001** (1 > 2, 3, 4)	2.20 ± 0.86	2.13 ± 1.13	2.13 ± 0.99	2.20 ± 1.15	.977
Global score	16.40 ± 6.27	13.15 ± 5.70	12.35 ± 6.45	11.70 ± 5.91	<.001*** (1 > 2, 3, 4)	12.87 ± 6.94	11.13 ± 5.17	11.60 ± 5.30	11.40 ± 5.67	.513

**Table 4:** Changes of PSQI of the SR device group and the active control group during follow-up.

	Group A					Group B				
(M ± SD)	Baseline	4 wks	8 wks	12 wks	*P* value (post hoc)	Baseline	4 wks	8 wks	12 wks	*P* value
Personal subjective sleep quality	2.10 ± 0.64	1.55 ± 0.51	1.30 ± 0.47	1.45 ± 0.60	<.001*** (1 > 2, 3, 4)	1.53 ± 0.83	1.67 ± 0.82	1.60 ± 0.83	1.53 ± 0.83	.383
Sleep latency	1.85 ± 1.09	1.50 ± 0.95	1.30 ± 0.98	1.30 ± 1.03	.008** (1 > 2, 3, 4)	1.53 ± 1.13	1.53 ± 1.25	1.47 ± 1.19	1.47 ± 1.19	.915
Sleep hours	1.30 ± 0.86	1.40 ± 0.82	1.25 ± 0.79	1.35 ± 0.75	.633	1.20 ± 1.08	1.27 ± 1.10	1.20 ± 1.01	1.13 ± 0.99	.616
Sleep efficiency	1.55 ± 1.15	0.95 ± 0.89	0.85 ± 0.81	0.80 ± 0.77	<.001*** (1 > 2, 3, 4)	1.27 ± 1.28	1.13 ± 1.13	1.20 ± 1.15	1.07 ± 1.16	.467
Sleep trouble	1.35 ± 0.11	1.15 ± 0.08	1.20 ± 0.09	1.15 ± 0.08	.219	1.20 ± 0.11	1.27 ± 0.15	1.20 ± 0.11	1.27 ± 0.12	.820
Use of sleeping pills	0.45 ± 1.00	0.55 ± 0.94	0.30 ± 0.73	0.40 ± 0.88	.230	0.60 ± 1.06	0.33 ± 0.82	0.53 ± 1.06	0.40 ± 0.91	.270
Daytime dysfunction	0.85 ± 0.75	0.90 ± 0.55	0.75 ± 0.55	0.85 ± 0.59	.663	0.60 ± 0.51	0.80 ± 0.68	0.60 ± 0.63	0.60 ± 0.51	.402
Total score	9.45 ± 3.97	8.00 ± 2.94	6.95 ± 3.20	7.30 ± 3.31	<.001*** (1 > 3, 4)	7.93 ± 4.17	8.00 ± 4.64	7.80 ± 4.83	7.47 ± 4.55	.705

After 12 weeks of treatment, no obvious side effects were observed in either group.

## 4. Discussion

This study is the first to use the EMF as an add-on in the treatment and management of sleep problems of patients with nocturia and to examine its effectiveness and tolerability. Besides the study design being randomized but not placebo-controlled, it also has some other limitations. First, the sample size is small. Second, no objective measurements were used for evaluation, such as polysomnography (PSG) or actigraphy for sleep, and urodynamic study, and the placebo effect cannot be ruled out. Third, adherence to the medication could not be verified, but we confirmed that the sleep device was turned on every night. Last, we did not classify the types or etiologies of nocturia. Due to these limitations, we need to be cautious in interpreting findings of this study.

Thirty-five participants with nocturia completed the study process, with most of them male and aged 50 to 70 years old. They were relatively healthy since we excluded patients with major diseases, although approximately 20% to 30% had hypertension or diabetes. The SR sleep device was well tolerated among our participants. During the recruitment and follow-up, none dropped out due to adverse effects, although 2 participants could not return for follow-up as scheduled and were thus excluded. Our findings were similar to our previous study using the SR sleep device in patients with primary insomnia, with very few participants experiencing side effects (headache and dizziness, one in the study group and one in the placebo group) (Y.S. Huang, I. Tan, W. Chin, et al. unpublished data).

Patients with nocturia responded well to the SR sleep device. Individuals in group A saw their sleep condition (PSQI and ESS) significantly improved, but those in group B did not (Table 2; Fig. 3C and D). Further analysis of PSQI revealed that subjective sleep quality and sleep efficiency of the SR sleep device group significantly improved during follow-up (Table 4). Our previous study of the SR sleep device also showed that the SR sleep device group experienced significant improvement not only in subjective sleep quality, sleep latency, sleep duration, and daytime sleepiness (PSQI and ESS) but also in sleep onset latency and total sleep by objective PSG (Y.S. Huang, I. Tan, W. Chin, et al. unpublished data). The results confirmed that the SR can be beneficial in sleep, and its implementation can include both primary and secondary sleep disorders.

Interestingly, the symptoms of nocturia and quality of life (AUASS and N-QOL) also improved significantly in the SR sleep device group. Further analysis of AUASS revealed that streaming and sleeping disturbed by nocturia were improved after 4-week intervention (Table 3). Despite trends toward improvement, the active control group did not demonstrate significant change. Oxybutynin can be used to treat overactive bladder and is commonly prescribed in clinical settings. However, it was not very effective in treating nocturia symptom in this study. Some symptoms improved but not significantly after treatment with oxybutynin, and it may be attributed to various reasons. The etiology of nocturia can differ due to age^[24]^ and limit the treatment effect of antimuscarinic medications. Younger patients may have reduced bladder capacity while urine production is increased in older patients. Only desmopressin has been proven to efficiently treat nocturia due to nocturnal polyuria. The evidence supporting other medications such as antimuscarinics, α1-blockers, and antiinflammatory medications in treating nocturia is weak to low.^[25]^ Drug compliance is another important issue. Considering the negative impacts of nocturia, more treatment options are needed.

No evidence is currently available to support the use of EMF in treating nocturia. However, several studies on benign prostate hyperplasia (BPH) and pulsed electromagnetic field (PEMF) have reported positive findings.^[26–28]^ PEMF can suppresses pain and inflammation and induce angiogenesis, as well as potentially affect the inflammatory process development in BPH.^[29]^ Xenophon et al found that, compared with α-blockers, patients with BPH receiving PEMF demonstrated significant improvement of BPH symptoms, including nocturia, as well as reduction in prostate volume, urine residue volume, and increased mean urine flow rate. A recent observational prospective study of Brardi et al^[30]^ reported a new device that generated an intense variable magnetic field and vibratory stimulation when applied at the perineal level. While only 10 males were not satisfied after conventional therapy, they found that nocturia, hesitancy, incomplete emptying, and urgency were all improved within the first week of treatment. Since BPH is a common cause of nocturia, improving nocturia with EMF can be related to its possible treatment effect of BPH.

Another possible mechanism of the effects of EMF on nocturia is via decreasing depression and anxiety symptoms in patients with nocturia. Depression is a nonurological cause of nocturia,^[31]^ and previous studies have shown that changes in geomagnetic activity may influence the incidence of depression.^[32]^ One study used transcranial PEMF as an add-on for 52 patients with moderate-to-severe depression, who reported significant improvement in their scores on the Hamilton depression scale.^[33]^ The SR sleep device has also been found to decrease patients’ sympathetic tone, as evaluated by heart rate variation analysis,^[34]^ and may also alleviate anxiety. However, further research is warranted to investigate the possible role and mechanism of the EMF and the SR sleep device in the treatment of nocturia.

Currently, the mechanisms of EMF and SR in sleep and nocturia are not fully understood. Cherry^[16]^ suggested that SR could be the possible biological mechanism that explains biological and human health effects of geomagnetic activity and may thus have a full impact for humans. Human brain waves and SR share the same frequency range, and the human body can also absorb and respond to natural EMF. Such natural EMF as SR can influence cell-to-cell communication in the human body,^[35]^ and Wang et al^[14]^ indicated that extremely low-frequency magnetic stimulation could induce low-frequency activities and resonance effects of the human brain. These biophysical mechanisms may explain biological and human health effects of geomagnetic activity and possible mechanisms of the SR sleep device. Double-blind, randomized placebo-controlled studies with objective measurements including blood sampling, prostate echo and sleep studies can further explore possible pathophysiological mechanisms of SR in improving nocturia.

## 5. Conclusions

This study revealed that the EMF as an add-on can have the potential to improve not only sleep and quality of life but also nocturia symptom in patients with nocturia. The SR sleep device group that received both oxybutynin and the SR sleep device experienced significant improvement in AUASS, N-QOL, PSQI, and ESS, but no significant change in these questionnaires was found in the active control group, which received medication only. In addition to primary insomnia, our results suggested that EMF can also be effective for sleep disturbances secondary to physical disease, which can be a new field of research for clinical application. In the future, studies with larger sample sizes and objective measurements can help to confirm our findings.

## Acknowledgments

We want to thank psychologist I Tang for data analysis.

## Footnotes

* *P* < .05,

***P* < .01,

*** *P* < .001.

* *P* < .05,

** *P* < .01,

*** *P* < .001.

** *P* < .01,

*** *P* < .001.

## References

[1] Van Kerrebroeck P Abrams P Chaikin D. The standardization of terminology in nocturia: report from the standardization subcommittee of the International Continence Society. BJU Int. 2002;90:11–5. 12445092 10.1046/j.1464-410x.90.s3.3.x

[2] Bosch JL Weiss JP. The prevalence and causes of nocturia. J Urol. 2010;184:440–6. 20620395 10.1016/j.juro.2010.04.011

[3] Schatzl G Temml C Schmidbauer J. Cross-sectional study of nocturia in both sexes: analysis of a voluntary health screening project. Urology. 2000;56:71–5. 10869627 10.1016/s0090-4295(00)00603-8

[4] Breyer BN Shindel AW Erickson BA. The association of depression, anxiety and nocturia: a systematic review. J Urol. 2013;190:953–7. 23680309 10.1016/j.juro.2013.03.126 PMC4153377

[5] Ancoli-Israel S Bliwise DL Norgaard JP. The effect of nocturia on sleep. Sleep Med Rev. 2011;15:91–7. 20965130 10.1016/j.smrv.2010.03.002 PMC3137590

[6] Yoshimura K Terai A. Classification and distribution of symptomatic nocturia with special attention to duration of time in bed: a patient-based study. BJU Int. 2005;95:1259–62. 15892812 10.1111/j.1464-410X.2005.05515.x

[7] Marshall SD Raskolnikov D Blanker MH. Nocturia: current levels of evidence and recommendations from the international consultation on male lower urinary tract symptoms. Urology. 2015;85:1291–9. 25881866 10.1016/j.urology.2015.02.043

[8] Johnson TM Vaughan CP Goode PS. Pilot results from a randomized trial in men comparing alpha-adrenergic antagonist versus behavior and exercise for nocturia and sleep. Clin Ther. 2016;S0149-2918:30742–1. 10.1016/j.clinthera.2016.10.001 28029383

[9] Edinger JD Arnedt JT Bertisch SM. Behavioral and psychological treatments for chronic insomnia disorder in adults: an American Academy of Sleep Medicine systematic review, meta-analysis, and GRADE assessment. J Clin Sleep Med. 2021;17:63–298. 10.5664/jcsm.8988 PMC7853211 33164741

[10] Belisheva NK Popov AN Petukhova NV. Qualitative and quantitative evaluation of the effect of geomagnetic field variations on the functional state of the human brain. Biophysics. 1995;40:1014–7. 8555283

[11] Mulligan BP Suess-Cloes L Mach QH. Geopsychology geophysical matrix and human behaviour. Man and the Geosphere. Nova Science Publishers 2010:115–141.

[12] Ghione S Mezzasalma L Del-Seppia C. Do geomagnetic disturbances of solar origin affect arterial blood pressure. J Hum Hypertens. 1998;12:749–54. 9844945 10.1038/sj.jhh.1000708

[13] Burch JB Reif JS Yost MG. Geomagnetic disturbances are associated with reduced nocturnal secretion of a melatonin metabolite in humans. Neurosci Lett. 1999;266:209–12. 10465710 10.1016/s0304-3940(99)00308-0

[14] Wang CX Hilburn IA Wu DA. Transduction of the geomagnetic field as evidenced from alpha-band activity in the human brain. eNeuro. 2019;6:1–23. 10.1523/ENEURO.0483-18.2019 PMC6494972 31028046

[15] Sentman DD. Schumann Resonances. Volland H, editor. In: Handbook of Atmospheric Electrodynamics. Boca Raton, FL: CRC Press 1995:267–298.

[16] Cherry R. Schumann resonances, a plausible biophysical mechanism for the human health effects of solar/geomagnetic activity. Nat Hazards. 2002;26:279–331.

[17] Persinger MA. Schumann resonance frequencies found within quantitative electroencephalographic activity: implications for earth-brain interactions. Int Lett Chem. 2014;11:24–32.

[18] Jirschele K Sand PK. Oxybutynin: past, present, and future. Int Urogynecol J. 2013;24:595–604. 22976530 10.1007/s00192-012-1915-8

[19] Buysse DJ Reynolds CF Monk TH. The Pittsburgh sleep quality index: a new instrument for psychiatric practice and research. Psychiatry Res. 1989;28:193–213. 2748771 10.1016/0165-1781(89)90047-4

[20] Tsai PS Wang SY Wang MY. Psychometric evaluation of the Chinese version of the Pittsburgh Sleep Quality Index (CPSQI) in primary insomnia and control subjects. Qual Life Res. 2005;14:1943–52. 16155782 10.1007/s11136-005-4346-x

[21] Johns M W. A new method for measuring daytime sleepiness: the Epworth sleepiness scale. Sleep. 1991;14:540–5. 1798888 10.1093/sleep/14.6.540

[22] Barry MJ Fowler FJ Jr O’Leary MP. Measurement committee of the American Urological Association. The American Urological Association symptom index for benign prostatic hyperplasia. J Urol. 1992;148:1549–57. 1279218 10.1016/s0022-5347(17)36966-5

[23] Hunt SM McEwen J McKenna SP. Measuring health status: a new tool for clinicians and epidemiologists. J R Coll Gen Pract. 1985;35:185–8. 3989783 PMC1960139

[24] Weiss JP Blaivas JG Jones M. Age related pathogenesis of nocturia in patients with overactive bladder. J Urol. 2007;178:548–51. 17570424 10.1016/j.juro.2007.03.117

[25] Cornu JN Abrams P Chapple CR. A contemporary assessment of nocturia: definition, epidemiology, pathophysiology, and management—a systematic review and meta-analysis. Eur Urol. 2012;62:877–90. 22840350 10.1016/j.eururo.2012.07.004

[26] Elgohary HM Tantawy SA. Pulsed electromagnetic field with or without exercise therapy in the treatment of benign prostatic hyperplasia. J Phys Ther Sci. 2017;29:1305–10. 28878453 10.1589/jpts.29.1305 PMC5574357

[27] Giannakopoulos XK Giotis C Karkabounas SC. Effects of pulsed electromagnetic fields on benign prostate hyperplasia. Int Urol Nephrol. 2011;43:955–60. 21537858 10.1007/s11255-011-9944-7

[28] Tenuta M Tarsitano MG Fattorini G. Pulsed electromagnetic field (PEMF) therapy effects on human prostate volume in the treatment of benign prostatic hyperplasia (BPH). Maturitas. 2019;124:175.

[29] Johnson MT Waite LR Nindl G. Noninvasive treatment of inflammation using electromagnetic fields: current and emerging therapeutic potential. Biomed Sci Instrum. 2004;40:469–74. 15134003

[30] Brardi S Biandolino P Giovannelli V. Possible applications of electromagnetic fields in the treatment of symptoms related to benign prostatic hyperplasia. Am J Urol Res. 2020;5:006–10.

[31] Weiss JP. Nocturia: focus on etiology and consequences. Rev Urol. PMC3602727 23526404

[32] Kay RW. Geomagnetic storms: association with incidence of depression as measured by hospital admission. Br J Psychiatry. 1994;164:403–9. 8199794 10.1192/bjp.164.3.403

[33] Larsen ER Licht RW Nielsen RE. Transcranial pulsed electromagnetic fields for treatment-resistant depression: a multicenter 8-week single-arm cohort study: the eighth trial of the Danish University Antidepressant Group. Eur Psychiatry. 2020;63:e18. 32093804 10.1192/j.eurpsy.2020.3 PMC7315871

[34] Tang I. A study on the improvement of symptoms of insomnia patients with Schumann wave device from the factors related to cognitive hyperarousal and distress (Master’s thesis, Fu Jen Catholic University, Taipei, Taiwan), 2020. Available at: https://hdl.handle.net/11296/f529aa.

[35] Cherry NJ. Human intelligence: the brain, an electromagnetic system synchronised by the Schumann Resonance signal. Med Hypotheses. 2003;60:843–4. 12699709 10.1016/s0306-9877(03)00027-6

